# A Case Report Based on a Holistic Approach to Improve Assisted Reproductive Technology (ART) Pregnancy Outcomes in a Patient With Thyroid Dysfunction

**DOI:** 10.7759/cureus.53144

**Published:** 2024-01-29

**Authors:** Neha Nawale, Akash More, Shilpa Dutta, Namrata Choudhary, Sanket Mahajan, Shivani Khemani

**Affiliations:** 1 Clinical Embryology, School of Allied Health Science, Datta Meghe Institute of Higher Education and Research, Nagpur, IND; 2 Clinical Embryology, Wardha Test Tube Baby Centre, Wardha, IND

**Keywords:** icsi, yog nidra, yoga, hypothyroidism, infertility

## Abstract

To address infertility in a 34-year-old woman with hypothyroidism, this case study investigated an integrated holistic therapy approach. The woman presented with primary infertility and sought treatment at an infertility centre in Maharashtra, India. She underwent thorough evaluations for infertility, revealing a history of hypothyroidism. The therapy strategy included a six-month course of 50 mg levothyroxine, along with yoga and yog nidra. The yoga interventions, incorporating techniques such as surya namaskar, kriyas, yoga asanas, and pranayama, proved beneficial for weight management, stress reduction, and overall well-being. The deep relaxation method known as yog nidra played a crucial role in achieving hormonal balance. After six months, the patient's body mass index (BMI) improved from 28 to 24, and the male partner's semen analysis showed results within normal limits. The patient's thyroid-stimulating hormone (TSH) levels also returned to the normal range. Further in-vitro fertilization (IVF) treatment, including a successful embryo transfer, resulted in a positive clinical pregnancy test. This research underscores the effectiveness of alternative therapies like yoga and meditation in conjunction with traditional medicine to address both the psychological and physiological components of infertility caused by hypothyroidism. The case suggests that an integrated approach like this could offer a comprehensive solution for infertile couples. However, for broader applicability, additional investigation and clinical validation are warranted.

## Introduction

Over 186 million individuals and 48 million couples experienced infertility globally [1,2]. For more than three years, assisted reproductive technologies (ART) were available, with ART interventions such as in-vitro fertilization (IVF). More than five million of children were born as a result [1,2]. Deficiency of thyroid hormone was a frequent medical condition known as hypothyroidism. If hypothyroidism goes untreated, it may have major adverse effects on health and could even cause death. A common indicator of early thyroid failure was mild or subclinical hypothyroidism, characterized by thyroid-stimulating hormone (TSH) levels and free thyroxine levels within the normal range [3]. The thyroid could affect fertility in different ways, including luteal phase defects, increased prolactin levels, and sex hormone abnormalities. Therefore, healthy thyroid function was essential for fertilization. It could also lead to adverse effects on the menstrual cycle, producing irregular ovulation and lowering the quality of eggs. To increase the chances of conception, thyroid levels must be controlled under medical supervision. Blood TSH levels make it easy to identify hypothyroidism. Subclinical hypothyroidism was indicated by slightly increased TSH levels, along with normal triiodothyronine (T3) and thyroxine (T4) levels, while clinical hypothyroidism was indicated by increased TSH decreased T3 and T4 levels [4]. Levothyroxine treatment for hypothyroidism typically improves fertility, reverses hormonal changes, and returns menstruation to normal. However, some women who have had their hypothyroidism treated still experience infertility and turn to IVF or other treatments [5].

The integrated approach to yoga therapy (IAYT) is a comprehensive and integrated strategy that combines different yoga practices at every level to treat a range of illnesses and their associated symptoms. Numerous health issues, including obesity, stress, and depression, that are linked to hypothyroidism have been shown to improve with yoga practice [6]. When compared to sleep, yog nidra aims to induce a deep level of relaxation in which the practitioner is still aware of their surroundings. It is said that yog nidra helps to induce positive changes in both mental and physical health. The main stages of yog nidra relaxation are outlined, along with some new experimental results on the physiological and psychological impacts of the practice [7].

## Case presentation

Patient information

An infertile couple visited the infertility clinic in Maharashtra, India. The couple, a 34-year-old female and a 38-year-old male, presented with a complaint of primary infertility for the past year. They were provided with a detailed explanation of the processes, benefits, and drawbacks. Informed consent was obtained from both individuals.

Medical/surgical history

The couple reportedly had no history of surgical interventions. The male had no medical history, while the female had a clinical history of hypothyroidism. This was their initial visit for infertility treatment.

Physical examination

The body mass index (BMI) of the female was 28 kg/m^2^, while that of the male was 22 kg/m^2^. According to the physical examination, the female was overweight, whereas the husband was within the normal range.

Investigation

To determine the underlying cause of their infertility, both underwent extensive assessments.

After analyzing the husband's semen report, the count was 89 million/mL, motility was 80%, and the normal morphology was 5%. According to the investigation, the report was normal.

The female patient underwent a thyroid function test; according to the report, the level of serum TSH was 7.1 mIU/L, T3 was 0.5 nmol/l, T4 was 60 nmol/l, and anti-Müllerian hormone (AMH) was 0.6 ng/mL. Table 1 mentions the before and after values of the blood serum parameters analysed for thyroid function.

**Table 1:** Before and after values of TSH, T3 and T4. TSH: thyroid stimulating hormone, T3: triiodothyronine, T4: thyroxine

PARAMETERS	BEFORE VALUE	AFTER VALUE	REFERENCE VALUE
TSH Value mIU/L	7.1 mIU/L	3.6 mIU/L	0.4-4.0 mIU/L [8]
T3 Value nmol/l	0.5 nmol/l	2.0 nmol/l	1.2-2.8 nmol/l [8]
T4 Value nmol/l	60 nmol/l	100 nmol/l	77-155 nmol/l [8]

Treatment

The patient was advised to take a 50 mg levothyroxine tablet along with yoga and yog nidra. Yoga aids in weight loss and enhances blood circulation, whereas yog nidra helps alleviate stress. We administered this treatment for a total of six months.

Yogic Interventions

**Figure 1:**
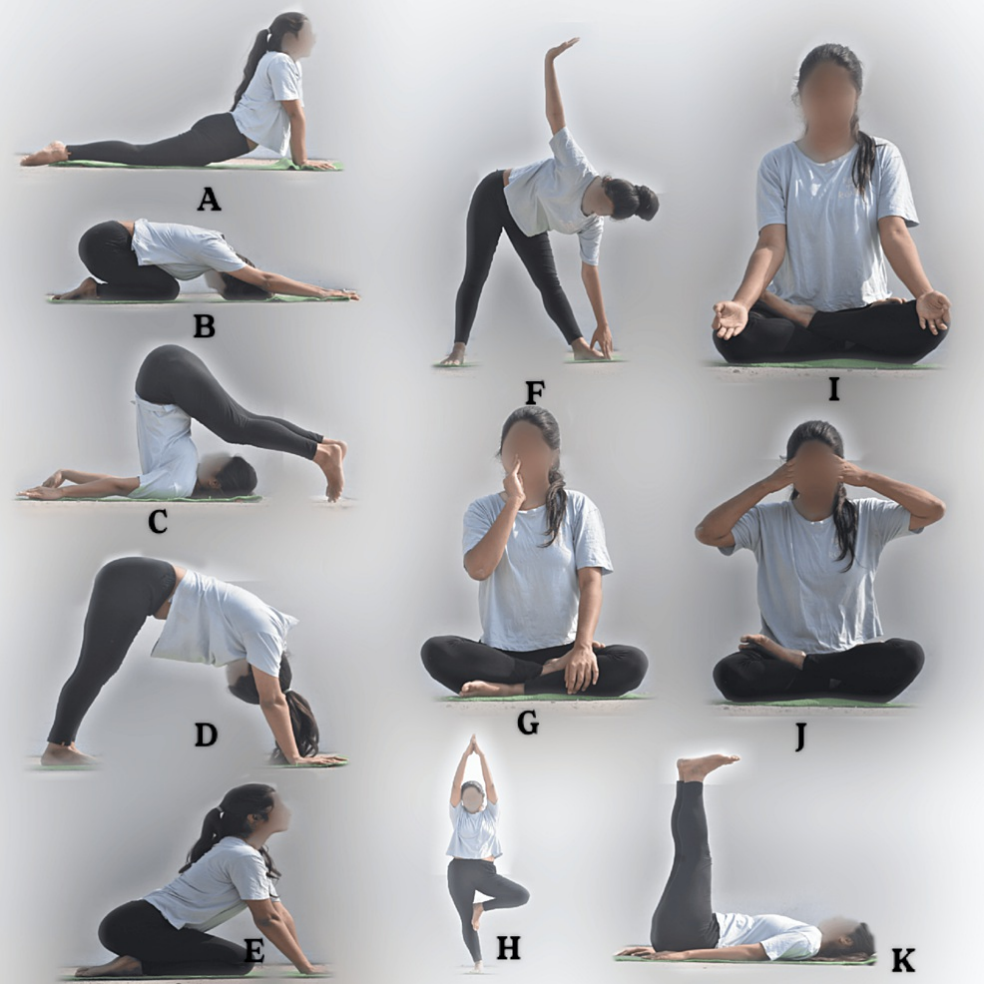
Yoga positions advised to the patient for treatment. A - Bhujangasana, B - Adho Mukha Virasana, C - Halasana, D - Adho Mukha Svanasana, E - Simhasana, F - Trikonasana, G - Anulom Vilom, H - Vrikshasana, I - Ujjayi Pranayama, J - Bhramari Pranayama, K - Viparita Karani The original picture was clicked by the author of this article and informed consent was obtained for publication from the patient.

The patient was prescribed a yoga regimen consisting of four sets of surya namaskar, each of which was followed by a deep relaxation exercise. Weekly modifications to the routine included particular cleansing kriyas like vamana, which purifies the digestive system, jalneti, which cleans the upper respiratory tract, and sutra neti, which opens the upper nasal tract. Table 2 lists the suggested yoga positions to help with hypothyroidism, and Figure 1 shows the corresponding poses. The entire yoga session lasted 18 minutes, with a focus on a holistic approach to improve mental and physical health.

**Table 2:** Details of yogasana advised to the patient.

NAME OF YOGASANA	DURATION	NO. OF CYCLES
Viparita Karani	1 minute	1 Cycle
Sarvangasana	2 minutes	2 Cycles
Matsyasana	2 minutes	3 Cycles
Vrikshasana	2 minutes	1 Cycle
Bhujangasana	1 minute	1 Cycle
Setu Bandasana	2 minutes	2 Cycles
Dhanurasana	2 minutes	3 Cycles
Trikonasana	1 minute	1 Cycle
Simhasana	2 minutes	3 Cycles
Halasana	2 minutes	2 Cycles
Adho Mukha Virasana	1 minute	1 Cycle

A daily routine of 15 minutes of pranayama, combining various breathing techniques to improve both physical and mental health, may significantly enhance overall well-being. Anulom-vilom pranayama, focusing on breathing via alternating nostrils for two minutes, and ujjayi pranayama, involving deep, controlled breathing for two minutes, was performed. Additionally, a two-minute fast and vigorous breath exercise known as kapalabathi, which increases lung capacity, was executed. The practice also included four breathing exercises (Brahmari, Bhastrika, Suryabhedi, and Nadi Shodhana), each taking two minutes, addressing different areas of cleansing and breath control. Following pranayama, a daily meditation practice for five minutes promotes relaxation and mental clarity. Moreover, practising yog nidra, a type of music therapy for sleep, every day facilitated significant achievements.

After six months of yoga and meditation treatment, the patient revisited the IVF clinic and was advised to undergo a TSH test, which showed 3.5 mU/L, T3 2.5 nmol/l, and T4 100 nmol/l. The report also indicated a BMI of 24kg/m^2^. On day 13 of the menstrual cycle, the patient was triggered for follicular aspiration. Thirty-six hours after the trigger, ovum pickup was scheduled, during which seven oocytes were retrieved (3 MII, 2 MI, 2 MII). Subsequently, intracytoplasmic sperm injection (ICSI) was performed. On day 5, two blastocysts were formed, and embryo freezing was carried out. After two months, embryo transfer was scheduled, and the embryos were thawed. The quality of the embryos was 4AB and 3AA. After a few hours, the embryos were transferred.

Follow-up

The patient had been advised to take regular medication along with yoga therapy after being discharged from the embryo transfer. The result of testing a female blood sample for serum human chorionic gonadotropin (β-hCG) after 14 days of embryo transfer was 805 mIU/mL. We advised the patient to visit clinics on a regular schedule for follow-up after a successful pregnancy.

## Discussion

Thyroid diseases are among the most common endocrine problems in women, often causing infertility. In the complex origin of infertility, thyroid problems, specifically hypothyroidism, are frequently identified as the leading cause. Women experiencing infertility may also undergo psychological and emotional stress [9]. In our case report, the female suffered from hypothyroidism, presenting with an increased BMI and elevated thyroid levels.

Infertility is often linked to thyroid dysfunction, which can be effectively treated by restoring appropriate levels of thyroid hormones. Hormone therapy with thyroxine is the most common treatment for established hypothyroidism. TSH measurements were taken every six to eight weeks to assess medication compliance and effectiveness [4]. Following a similar approach with our patient, we instructed her to take levothyroxine for six months and monitored her TSH, T3, and T4 levels every four weeks to assess their levels.

Research has indicated that yoga intervention can positively impact serum thyroid hormone (sTSH) levels in hypothyroid women. After six months of yoga intervention, Neelakanthan et al. (2016) observed a significant decrease in BMI, improvements in lipid profiles, and reduced TSH levels. Other studies on women with hypothyroidism showed enhancements in pulmonary function tests and quality of life following yoga intervention. The findings of our study align with previous research on the benefits of yoga for depression, including improved mood, cognitive abilities, and serum cortisol levels [10]. Similarly, our patient experienced significant reductions in BMI and TSH levels after six months of yoga therapy.

According to Sowjanya Dumbala et al., yoga therapy has a positive impact on the psychological discomfort experienced by infertile women undergoing treatment. Anxiety levels improved in all studies reviewed, with women receiving IVF therapy reporting lower levels of anxiety and despair after participating in yoga intervention. Six weeks of structured yoga therapy resulted in a 20% reduction in state anxiety and a 12% decrease in trait anxiety scores [11]. We recommended yoga therapy to our patient and observed positive psychological changes after six months of therapy. The incorporation of suryanamaskar improved infertility issues and reduced negative thoughts.

Yog nidra considered one of the best methods for both mental and physical relaxation, as well as preparing the mind for yogic discipline, significantly contributed to our treatment plan. Despite its association with relaxation-meditation practices, yog nidra differs significantly from both seated meditation and ordinary relaxation [7]. In our case, yog nidra significantly aided in hormonal balance during the course of our six-month treatment, proving beneficial for hypothyroidism.

## Conclusions

The case study emphasizes the necessity for careful management of hypothyroidism, a common cause of infertility in women. Combining yoga and yog nidra with levothyroxine medication was demonstrated to be an effective strategy for addressing both the psychological and physiological components of infertility. Yoga therapies, including specific asanas and pranayama, assisted individuals in managing their weight, reducing stress, and enhancing their overall well-being. According to our case report, couples grappling with infertility may find comprehensive resolution through an integrated approach that combines medical care with complementary therapies such as yoga and meditation.
